# Broken-Symmetry Quantum Hall States in Twisted Bilayer Graphene

**DOI:** 10.1038/srep38068

**Published:** 2016-12-01

**Authors:** Youngwook Kim, Jaesung Park, Intek Song, Jong Mok Ok, Younjung Jo, Kenji Watanabe, Takashi Taniquchi, Hee Cheul Choi, Dong Su Lee, Suyong Jung, Jun Sung Kim

**Affiliations:** 1 Department of Physics, Pohang University of Science and Technology, Pohang 37673, Korea; 2 Korea Research Institute of Standards and Science, Daejeon 305-340, Korea; 3 Center for Artificial Low Dimensional Electronic System, Institute for Basic Science (IBS), Pohang 37673, Korea; 4 Department of Chemistry, Pohang University of Science and Technology, Pohang 37673, Korea; 5 Department of Physics, Kyungpook National University, Daegu 702-701, Korea; 6 National Institute for Materials Science, 1-1 Namiki, Tsukuba 305-0044, Japan; 7 Applied Quantum Composites Research Center, KIST Jeonbuk Institute of Advanced Composite Materials, Jeonbuk 55324, Korea

## Abstract

Twisted bilayer graphene offers a unique bilayer two-dimensional-electron system where the layer separation is only in sub-nanometer scale. Unlike Bernal-stacked bilayer, the layer degree of freedom is disentangled from spin and valley, providing eight-fold degeneracy in the low energy states. We have investigated broken-symmetry quantum Hall (QH) states and their transitions due to the interplay of the relative strength of valley, spin and layer polarizations in twisted bilayer graphene. The energy gaps of the broken-symmetry QH states show an electron-hole asymmetric behaviour, and their dependence on the induced displacement field are opposite between even and odd filling factor states. These results strongly suggest that the QH states with broken valley and spin symmetries for individual layer become hybridized via interlayer tunnelling, and the hierarchy of the QH states is sensitive to both magnetic field and displacement field due to charge imbalance between layers.

Bilayer two-dimensional electron gas system (2DES), a pair of 2DESs in close proximity, reveals various intriguing quantum Hall (QH) phenomena arising from the additional layer degree of freedom^1^,^2^,^3^,^4^,^5^,^6^. The rich quantum Hall physics in bilayer 2DESs originates from the interplay of several characteristic energies such as cyclotron energy, Zeeman splitting, intra- and interlayer Coulomb interactions, and the interlayer tunnel coupling. In particular, interlayer tunnelling allows the Landau level (LL) mixing, forming symmetric or antisymmetric QH states that can be tuned by the interlayer separation. The resulting symmetric-antisymmetric gap, Δ_SAS_, is comparable with interlayer Coulomb interaction and often leads to unusual QH states distinct from those in single layer 2DESs, including magnetic field driven collapse of the tunnelling gap^3^ and the presence of Bose-Einstein condensate states^1^,^2^,^3^,^4^,^5^,^6^.

Twisted bilayer graphene, two single layer graphene sheets stacked with an arbitrary angle of orientation, offers a different kind of the bilayer 2DESs. Unlike the conventional bilayer 2DESs based on semiconductor heterosturctures, the layer separation is extremely small (*d*~0.4 nm). The coherent interlayer coupling, however, is strongly suppressed by the momentum mismatch between two Dirac cones from each layer, separated in the momentum space. Although the merging of two Dirac cones of each layer at very small twist angles (*θ* < 2°) drastically alters the low-energy electronic structure^7^,^8^,^9^,^10^,^11^,^12^,^13^, in most cases with large twist angles the low energy states of the two layers are only tunnel-coupled, similar to the double quantum well in semiconductor heterostructures. This breakdown of interlayer coherence^14^ unties the layer degree of freedom from spin and valley counterparts for each layer, providing eight-fold degeneracy in the LLs. This contrasts to the case of zeroth LL in Bernal-stacked bilayer graphene where spin, valley and orbital degrees of freedom introduce the eight-fold degeneracy with the layer degree of freedom tied to the valley. Thus, twisted bilayer graphene offers an intriguing platform for studying the interaction-induced QH states in 2DES with multiple degrees of freedom.

In this work, we report experimental results on broken-symmetry QH states in high-quality twisted bilayer graphene with a large twist angle. We observed all the broken-symmetry QH states of the eight-fold zeroth LL; *ν*_tot_ = 0, ±1, ±2, and ±3, where *ν*_tot_ is the total filling factor of the bilayer system. The activation energies for the broken-symmetry QH states with even and odd filling factors show an opposite dependence on charge imbalance between the layers. The even-odd effect strongly suggests that the QH states for each layer with broken valley and spin symmetries become hybridized via interlayer tunnelling in twisted bilayer graphene. We have found that the hierarchy of the broken-symmetry QH states is sensitive to external magnetic field and internal displacement field between layers from charge imbalance.

## Results

### Device characterization and quantum Hall effect

A high-quality twisted bilayer graphene devices were fabricated by the so-called Van der Waals pick-up transfer technique^15^. The twist angle of the graphene layers (θ) was estimated to be ~5° for the device 1 (D1) and ~3° for the device 2 (D2) from Raman spectroscopy measurement^16^,^17^, as shown in the Supplementary Information. The energy of the saddle point with respect to the Dirac point is ~0.6 eV (D1) and ~0.3 eV (D2)^18^, which is far beyond the energy range accessible by the back-gate voltage (*V*_g_) modulation in our devices. Transport measurements were carried out using the conventional low-frequency AC lock-in method as a function of gate voltage at different magnetic fields (*B*) and temperatures (*T*).

**Figure 1:**
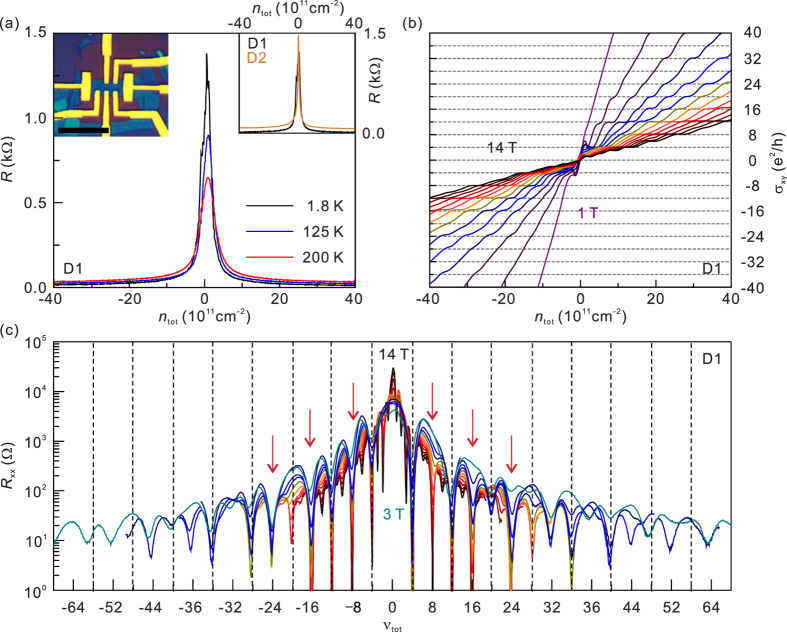
(**a**) Longitudinal resistance *R*_*xx*_ of D1 as a function of back-gate voltage *V*_g_ at various temperatures. The left inset is the optical image of D1 with a 10 μm scale bar. The right inset shows longitudinal resistance of D1 and D2 at 1.8 K. (**b**) The Hall conductivity *σ*_*xy*_ as a function of *n*_tot_ at *B* fields from 1 T to 14 T in 1 T steps for the D1. (**c**) *R*_*xx*_ of D1 as a function of the total filling factor *ν*_tot_ at different *B* fields from 3 T to 14 T in 1 T steps. The vertical dashed lines correspond to *ν*_tot_ = 2·*ν*_single_ = 2·(4*N* + 2) = 4, 12, 20, 28, 36,…, where *N* is the Landau index.

Figure 1(a) shows the curves of longitudinal resistance *R*_*xx*_ as a function of *V*_g_ at different temperatures. At *T* = 1.8 K, the charge neutral point is *V*_g_~0 V for both devices, and the Full-Width-at-Half-Maximum (FWHM) of the *R*_*xx*_ peak is ~3.0 V (D1) and ~1.5 V (D2). The corresponding residual charge density is ~2.0 × 10^11^ cm^−2^ (D1) and ~1.0 × 10^11^ cm^−2^ (D2). Carrier mobility in both devices is estimated to be larger than 50,000 cm^2^/V s at *n*~10^12^ cm^−2^ for electrons and holes. Signatures of Hall conductance (*σ*_xy_) plateaus at the integer multiples of *e*^2^/*h* was observed above *B* = 1 T, and the broken-symmetry QH states start to emerge at *B* = 9 T as shown in Fig. 1(b). In high filling factors of |*ν*_tot_| ≥ 4, we observed clear QH plateaus even for the *ν*_tot_ = ±16 states at a relatively low fields of 8 T. Note that in previous studies on twisted bilayer graphene^12^,^19^,^20^,^21^,^22^, the QH plateau was only visible for the *ν*_tot_ = 8 states under high magnetic field of 15 T. These observations confirm that our twisted bilayer graphene has high quality, comparable to previously reported high-quality monolayer and bilayer devices^23^,^24^,^25^,^26^,^27^,^28^,^29^,^30^.

### Quantum Hall state at high filling factors

**Figure 2:**
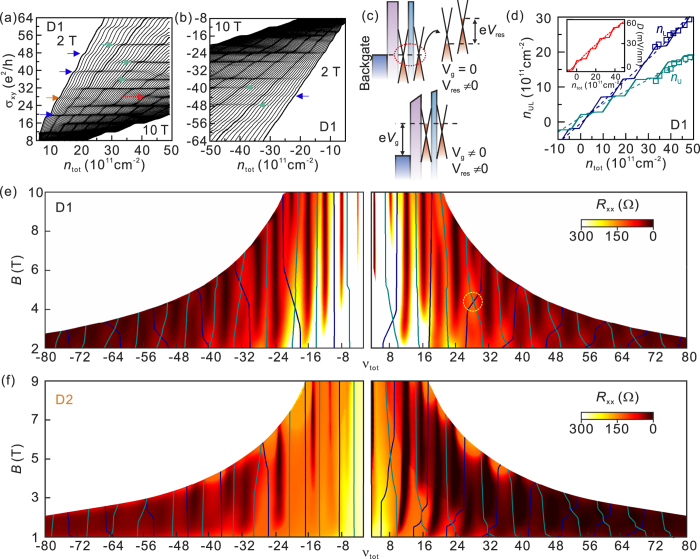
Hall conductivity *σ*_*xy*_ measured at *B* fields from 2 T to 10 T with a step of 0.10 T in (**a**) electron and (**b**) hole doping regimes. The blue arrows (green arrows) indicate the disappearance (reappearance) of the QH signatures. At *ν*_tot_ = 28, QH plateau disappears (orange arrow) and reappears (red arrow) with variation of total charge density *n*_tot_. (**c**) Schematic illustration of band alignment diagram for twisted bilayer graphene. Upper and lower panels describe the cases with zero and a finite gate voltage *V*_g_, respectively. Due to the residual charge density, residual electric field, e*V*_res_, is induced even at *V*_g_ = 0 as illustrated in the magnified right inset of the upper pannel. (**d**) Calculated carrier densities *n*_U_ and *n*_L_ for the upper (navy) and the lower (dark cyan) layers with variation of *n*_tot_ at *B* = 10 T. The dot lines correspond to the carrier densities of each layer at zero magnetic field. For comparison, experimentally measured *n*_U_ (navy square) and *n*_L_ (dark cyan square) from Shubnikov-de Hass oscillations are plotted together. The inset shows the displacement field (*D*) due to carrier imbalance between the layers as a function of *n*_tot_ at *B* = 10 T (solid line) and 0 T (dashed line). Colour rendition of *R*_*xx*_ as function of the total filling factor, *ν*_tot_ for (**e**) D1 and (f) D2. The lines represent the calculated position of the Landau levels from each layer (see the text).

At first we focus on the QH states with high filling factors, |*ν*_tot_| ≥ 4. Figure 1(c) shows *R*_*xx*_ as a function of total filling factor, *ν*_tot_ = *n*_tot_·*h*/*eB*, at different magnetic fields. Here, *n*_tot_ is total charge carrier density from both layers and *h* is Planck’s constant. Assuming that the twisted bilayer graphene consists of two independent monolayers, we would expect *R*_*xx*_ minima at *ν*_tot_ = 2 · *ν*_single_ = 2·(4 *N* + 2) = 4, 12, 20, 28, …. However, *R*_*xx*_ minima are observed at *ν*_tot_ = 4, 8, 12, 16, 20, 24, … as shown in Fig. 1(c), which look similar to that reported in Bernal-stack bilayer graphene. Despite this similarity of the overall QH sequence, the evolution of several QH states in twisted bilayer graphene is distinct from those in Bernal-stacked bilayer. In twisted bilayer graphene, we observed LL crossings with increasing *B*, where the QH sequence changes. For example, the QH plateau at *ν*_tot_ = 28 (marked with an orange arrow in Fig. 2(a)) disappears at *B*~4 T and re-emerges at *B*~6 T (marked with a red arrow). In addition, the QH plateaus at *ν*_tot_ = 40 and 48 (marked with blue arrows) disappear at *B*~4.8 T, while the QH plateaus at *ν*_tot_ = 36, 44 and 52 (marked with green arrows) appear at larger *B* fields. The similar LL crossings are also observed in the hole-doped region, as shown in Fig. 2(b). The QH plateau at *ν*_tot_ = −44 (marked with a blue arrow in Fig. 2(b)) disappears at *B*~3 T while QH plateaus at *ν*_tot_ = −40 and *ν*_tot_ = −48 (marked with green arrows) appear at higher fields.

We attribute these LL crossings to the charge carrier imbalance between two layers in the twisted bilayer graphene^20^. As illustrated in Fig. 2(c), charge carrier densities of the upper and lower layers are different under the back-gate field, because of the incomplete screening of the gate field by the lower layer. In such an imbalanced regime, the QH states of each layer are expected to behave independently, and the LLs of each layer are filled with different filling rates as *V*_g_ increases. For example, when the first LL of the lower layer is completely filled, the lower layer becomes incompressible, forcing next induced charges to fill the LL of the upper layer. But unlike the case of the lower layer, filling of the LL in the upper layer requires additional charging energy to be paid off. Therefore depending on the charging energy, as compared to the energy difference between the LLs of the lower layer, the next LL of the lower layer becomes compressible before or after the LL of the upper layer is completely filled. In this scheme, the filling factors of the QH states in each layer shift in a staircase pattern but with different rates and different step heights as shown in Fig. 2(d). The corresponding configurations of the LL filling for each step in Fig. 2(d) can be found in the Supplementary Information.

We performed numerical analysis of LL formations in twisted bilayer graphene as a function of *V*_g_^14^,^26^,^31^,^32^,^33^,^34^. Chemical potential difference between layers, 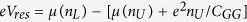, where *n*_U_ (*n*_L_) are carrier densities of the upper (lower) layers, e*V*_res_ is extrinsic electric field by residual charges, and *C*_GG_ is capacitance between the layers. We assumed the Lorentzian-shape density of states (DOS) for LLs with a peak broadening of 0.01 meV at *E*_*N*_ = sgn(*N*)*v*_F_(2*eħB*|*N*|)^1/2^. We used the interlayer dielectric constant *ε*_GG_ = 2.45*ε*_0_ (*ε*_0_ is the permittivity of vacuum)^31^ and the Fermi velocity of *v*_F_ = 0.85 × 10^6^ m/s (D1) and *v*_F_ = 0.75 × 10^6^ m/s (D2) that are estimated from Raman spectroscopy^33^. In order to reproduce the observed electron-hole asymmetry, we introduced *V*_res_ = 7 mV (4 mV) for D1 (D2), corresponding to the residual charges with a density of 2.0 × 10^11^ cm^−2^ (1.0 × 10^11^ cm^−2^) as obtained from Fig. 1(a). Figure 2(d) shows the calculated carrier density variations for upper (*n*_U_) and lower (*n*_L_) layers as a function of *V*_g_ at *B* = 10 T. As explained above, carrier densities for upper and lower layers exhibits a step-like increase at different rates with increasing *V*_g_. In real systems at a finite temperature, however, the LL broadening can be comparable with the LL spacing at high *V*_g_’s, and more monotonous *V*_g_-dependences are expected in *n*_U_ and *n*_L_, which is similar to the results for zero magnetic fields (dotted lines in Fig. 2(d)). This is indeed the case as experimentally confirmed by monitoring the Shubnikov-de Haas (SdH) oscillations (see the Supplementary Information). The measured *n*_U_ and *n*_L_ from the SdH oscillations in the high *V*_g_ region show good agreement with the calculated curves (dotted lines) in Fig. 2(d). These results suggest that the resultant displacement field (*D*) becomes larger at higher *V*_g_ as the charge imbalance between layers becomes stronger (see the inset of Fig. 2(d)).

This strong displacement field in the high *V*_g_ region is important for understanding the LL crossings in twist bilayer graphene. Figure 2(e) is the colour rendition of longitudinal resistance *R*_*xx*_ for D1 as a function of total filling factor and magnetic field. The QH states at *ν*_tot_ = 4, 8, 12, 16, 20, 24, … are indicated by the dark shades, and the overlaid solid lines indicate calculated filling-factor variations where each LL is half filled and *R*_xx_ reaches maximum. As displayed in Fig. 2(e), the calculated curves are well-matched to experimental observations. The QH states at *ν*_tot_ = 8 and *ν*_tot_ = 16, which are not expected in twisted bilayer graphene, turn out to be the combination of single-layer QH states with [*ν*_upper_,*ν*_lower_] = [2, 6] and [6, 10], respectively. The LL crossings are reproduced in the calculation as well. In the highly doped regime with a sufficiently large *D*, the crossover between the states with the same *ν*_tot_ but with different combinations of *ν*_upper_ and *ν*_lower_ is seen in the colour rendition. For example, the LL crossing for the *ν*_tot_ = 28 state seen in Fig. 2(a) occurs when the QH state of [*ν*_upper_, *ν*_lower_] = [14, 14] is transformed to that of [*ν*_upper_, *ν*_lower_] = [10, 18] upon increasing *V*_g_ as indicated with a yellow circle in Fig. 2(e). We also observed similar Landau level crossings on D2 and also good agreement with calculations as shown in Fig. 2(f), although the detailed features look different due to different Fermi velocity and residual density, as compared to those of D1. The observation of the level crossings suggest that QH states in twisted bilayer graphene can be considered as the combination of QH states from two monolayer graphene with charge imbalance.

### Broken-symmetry Quantum Hall state at low filling factors

**Figure 3:**
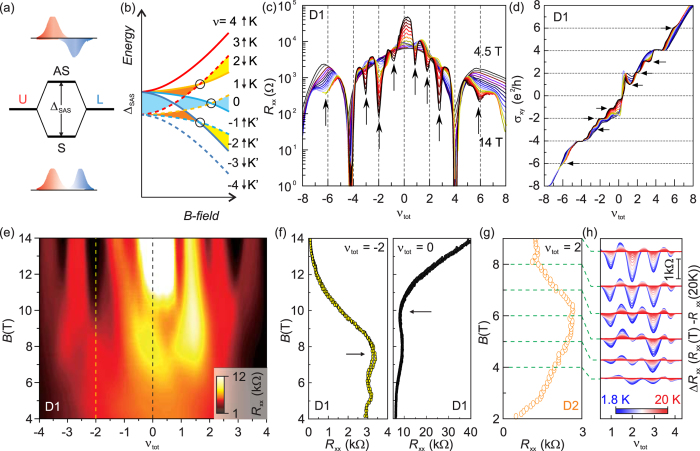
(**a**) Schematic illustration of symmetric-antisymmetric gap (Δ_SAS_) formation and the corresponding wave functions. AS(S) denotes the antisymmetric (symmetric) state, and U(L) denotes upper (lower) layer state. (**b**) Schematic drawing of the LL evolution for the broken-symmetry states with *B* field. The solid (dashed) line indicates the layer asymmetric (symmetric) levels. The valley (K, K’) and spin (↑, ↓) degrees of freedom are colour coded. The experimentally observed transitions are marked by circles. (**c**) The longitudinal resistance *R*_*xx*_ and (**d**) the Hall conductivity *σ*_*xy*_ as a function of total filling factor *ν*_tot_ at *B* fields from 4.5 T to 14 T with a step of 0.5 T for D1. The arrows indicate the *R*_*xx*_ dip and the corresponding Hall plateaus for the broken-symmetry QH state in zeroth and first landau levels. (**e**) Colour rendition of *R*_*xx*_ as a function of the total filling factor *ν*_tot_ for D1. Field-dependent *R*_*xx*_ (**f**) at *ν*_tot_ = −2 and 0 for D1 and (**g**) at *ν*_tot_ = 2 for D2 at 1.8 K. The *R*_*xx*_ dip and hump are marked by the arrows. (**h**) Temperature evolution of the longitudinal resistance, Δ*R*_*xx*_ (*R*_*xx*_(*T*) – *R*_*xx*_ (20 K)), from 1.8 K to 20 K as function of *ν*_tot_ at different magnetic fields for D2. The scale bar is 1 kΩ. The green dot lines between (**g**) and (**h**) indicate the corresponding magnetic fields from 4 T to 9 T in 1 T steps for data shown in (**h**).

Having understood the QH states with higher filling factors, we focus on the QH states with lower filling factors of |*ν*_tot_| < 4. These QH states are broken-symmetry QH states of the zeroth LLs where spin, valley, and layer degeneracies are fully lifted. In this regime, the displacement field is as low as |*D*| < 10 mV/nm and is not sufficient to induce the LL crossover between the normal integer QH states. At first, we notice that *R*_*xx*_ at the charge neutrality point, corresponding to the QH state at *ν*_tot_ = 0 QH state, increases with magnetic field (Fig. 3(a)). The behaviour is analogous to the quantum Hall insulator in single layer graphene^24^,^35^,^36^,^37^, where valley symmetry is broken before spin symmetry, resulting in the absence of the edge states. Thus, at high magnetic fields, the energy gap, developed by lifting valley degeneracy, remains larger than the gap from spin counterpart in our twisted bilayer device. In addition to *ν*_tot_ = 0 state, we identify other broken-symmetry QH states such as the even-integer states of *ν*_tot_ = ±2 above *B* = 8 T and the odd-integer states of *ν*_tot_ = ±1 and ± 3 above *B* = 11 T. Interesting is however that the electron-hole asymmetry appears between the broken-symmetry QH states, and the trend is opposite for even and odd fillings. As shown in Fig. 3(c), for the even filling factor states of *ν*_tot_ = ±2, the *R*_*xx*_ shows deeper valley at *ν*_tot_ = −2 than at *ν*_tot_ = 2. However, for the odd filling factors of *ν*_tot_ = ±1 or ±3, the QH states for electrons are more prominent than those for holes. This unusual electron-hole asymmetry suggests that charge imbalance between layers, either by external gate electric field or by remnant residual charges, significantly affects the formation of broken-symmetry QH states, similar to the cases of higher filling factors. We exclude the scenario that induced charges are continuously distributed between two layers for the broken-symmetry QH states since non-integer QH plateaus were not observed in *σ*_*xy*_.

The broken-symmetry QH states in twisted bilayer graphene can be qualitatively understood in terms of the layer-hybridized states with a finite interlayer coupling. In semiconductor double layer 2DES, each LL is four-fold degenerate; two from the spin and two from the layer degrees of freedom. While the former may be lifted by Zeeman splitting, the latter can be resolved by the interlayer tunneling which forms symmetric (S) and antisymmetric states (AS) out of the originally degenerate states. The splitting between the symmetric and antisymmetric states is parameterized by Δ_*SAS*_, which represents the tunneling strength between the layers. In twist bilayer graphene, a larger tunneling strength is expected than in conventional semiconductor heterostructures due to its small layer separation in an atomic length scale. Thus, for the layer-hybridized states in twisted bilayer graphene, the wave functions from the QH states of each layer having spin and valley degeneracy are mixed, forming the symmetric and antisymmetric states with a finite energy gap Δ_SAS_ as shown in Fig. 3(a). As illustrated in Fig. 3(b), the energy gaps of the broken-symmetry QH states with valley (K, K’) and spin (↑, ↓) have different dependence on magnetic field while Δ_SAS_ remains nearly independent on magnetic field. In this picture, transitions between the broken-symmetry QH states are possible at the same filling factor but with different combinations of broken symmetries among spin, valley and layer. For the QH state with *ν*_tot_ = 0 (blue shade in Fig. 3(b)), the corresponding low-*B* QH state is either the fully layer-symmetric state without spin and valley polarization or the intermediate state where electron and hole channels with opposite valley and spin degrees of freedom coexist before symmetries are fully broken at high *B*. For the QH state at *ν*_tot_ = ±2, the transition occurs between the valley-polarized and spin-unpolarized state (orange shade) and the full spin and valley-polarized state (yellow shade).

**Figure 4:**
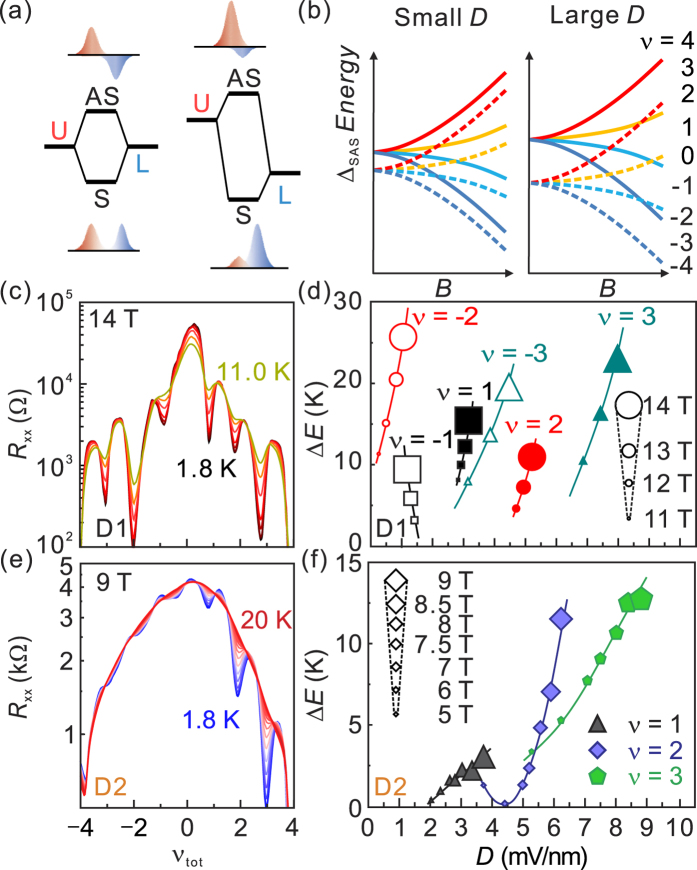
Schematic illustrations of (**a**) formation of symmetric-antisymmetric gap and (**b**) Landau level evolution with different displacement electric fields. With larger *D*-field, the energy gap for odd filling factors (*ν*_tot_ = ±1 and ±3) becomes bigger, while it becomes smaller for even filling factors (*ν*_tot_ = ±2) (**c**) *R*_*xx*_ of D1 taken at *B* = 14 T at various temperatures from 1.8 K to 11.0 K. (**d**) The energy gap of the QH states with *ν*_tot_ = −3, −2, −1, 1, 2, and 3, as a function of *D* field for D1. The symbol size corresponds to *B* fields of 10, 12 and 14 T, as presented in the right side. (**e**) *R*_*xx*_ of D2 taken at *B* = 9 T at various temperatures from 1.8 K to 20 K. (**f**) *D*-field dependent energy gap for the QH states with *ν*_tot_ = 1, 2, and 3 for D2. The symbol size shown in the left side indicates the applied magnetic fields from 5 T to 9 T.

We observe the signatures of these transitions. Following the vertical trace of *ν*_tot_ = −2 in Fig. 3(e), an *R*_*xx*_ hump is observed at *B*~7.5 T as indicated by the black arrow in Fig. 3(f). This is the transition from the valley-polarized and spin-unpolarized QH state at low *B* to the fully-polarized state at high *B*. We also identify the other transition for the QH state at *ν*_tot_ = 0 as the *R*_*xx*_ dip at *B*~10 T (black dotted line in Fig. 3(e) and (f)), which occurs when the intermediate broken-symmetry QH state is transformed into the full-symmetry-broken QH state at high *B*. A similar *R*_*xx*_ hump is also observed in the device 2 (D2) for the *ν*_tot_ = 2 state at *B*~5.5 T as shown in Fig. 3(g). In particular, D2 has a better device quality in the electron regime than D1, which allows us to investigate evolution of the *R*_*xx*_ minimum for the *ν*_tot_ = 2 state across the transition field *B*~5.5 T. In Fig. 3(h), we plot Δ*R*_*xx*_(*T*) = *R*_xx_(*T*) − *R*_*xx*_(20 K) as a function of the filling factor *ν*_tot_ at different magnetic fields for the broken-symmetry QH states at *ν*_tot_ = 1, 2, and 3. For the odd-integer QH states, *R*_*xx*_ minima become stronger with increasing magnetic field. In contrast, for the *ν*_tot_ = 2 state the *R*_*xx*_ minimum observed clearly at *B* = 4 T becomes weaker at *B* = 5–6 T, and eventually gets stronger above *B* = 7 T. The transport gap of the *ν*_tot_ = 2 state, estimated from the temperature dependence of *R*_*xx*_, indeed shows a minimum at *B*~6 T (Fig. 4(f)). These results confirm that the observed *R*_*xx*_ anomalies in Fig. 3(f) and (g) are a signature of transitions between the broken-symmetry QH states with different configurations of spin and valley degrees of freedom.

The remaining question is what causes the observed electron-hole asymmetric hierarchy of the broken-symmetry QH states shown in Fig. 3(c). We attribute this asymmetric behaviour to the interlayer displacement field introduced in the back-gated device. Depending on the relative strength of the displacement field, the energy difference between the QH states from each layer and thus the relative amplitude of the wave function of symmetric and antisymmetric QH state are varied (Fig. 4(a)). Accordingly the symmetric-antisymmetric gap Δ_SAS_ varies with the displacement field, which affects the size of transport gap Δ_*ν*_ measured at high *B.* In an ideal case without residual charges, the transport gaps should be identical between the QH states of electron-hole counterpart, *i.e.* Δ_*ν*_ = Δ_−*ν*_. In our devices, however, the displacement field is determined by both residual charges and the back-gate field. In D1, for example, the field induced by residual charges is compensated by the gate field at *V*_g_ ≈ −5 V, and near this gate voltage, the device is in the regime of *ν*_tot_ = −2 state at *B* > 10 T. Therefore we expect that the transport gap Δ_*ν*_ at *ν*_tot_ = −2 is much less affected by the displacement field than at *ν*_tot_ = +2.

The displacement field enhances Δ_SAS_, which shifts the four branches of the layer-symmetric states against those of the layer-asymmetric states as illustrated in Fig. 4(b). This shift eventually leads to opposite *D*-dependence of the transport gap Δ_*ν*_ of the even and odd filling factors. Figure 4(d) presents energy gap (Δ_*ν*_) of the D1 as a function of displacement field for different filling factors. We extracted Δ_*ν*_ from the temperature dependent *R*_*xx*_ measurements (Fig. 4(c)). For the transport gaps of *ν*_tot_ = ±2, Δ_*ν*_ is reduced by the presence of displacement field and thus, Δ_−2_ (weaker *D*) is always larger than Δ_2_ (stronger *D*). In contrast, Δ_*ν*_ of the odd-filling factor states such as *ν*_tot_ = ±1 and ±3 shows an opposite behaviour: Δ_*ν*_ increases gradually as the displacement field increases; Δ_3_ > Δ_−3_ > Δ_1_ > Δ_−1_ at a given magnetic field. In D2, we also observed similar trend. Although less clear *R*_xx_ minima of the QH states in the hole regime do not allow us to compare Δ_*ν*_ between electron and hole counterparts, we still observed the larger gap for the odd integer state as the displacement field increases; Δ_3_ > Δ_1_ as shown in Fig. 4(f).

## Discussion

Our observations clearly demonstrate that the interaction energies above *B* = 9 T follow the hierarchy of Δ_valley_ > Δ_spin_ > Δ_SAS_. This is in fact consistent with the energy scales estimated from previous studies^23^,^35^,^36^,^37^ on monolayer or twisted bilayer graphene. For monolayer graphene devices, electron-electron interaction lifts the valley and spin degeneracy and the corresponding energy scales are estimated to be ~100 K for the valley and 50~100 K for spin interaction at *B* = 10 T^23^,^35^,^36^,^37^. The energy scale for interlayer tunnelling in twisted bilayer graphene is found to be ~40 K^38^. Our study shows that the behaviour of the broken-symmetry QH states is very sensitive to the external magnetic field as well as the interlayer displacement field.

In conclusion, we investigate quantum Hall effects of the high-quality twisted bilayer graphene. Due to imperfect screening of the gate electric field, we find that measured QH signatures for high filling factors are due to combination of QH states independently formed at each layer, which is verified by the electrostatic model. At low filling factors, we observe broken-symmetry QH states and their transitions with the interplay of the relative strength of valley, spin and layer polarizations, which are sensitive to magnetic fields and the displacement field between the layers. These findings demonstrate that broken-symmetry QH states of twisted bilayer graphene can be magnetically and/or electrically tunable as in the cases of Bernal-stacked bilayer graphene. In addition, the interlayer coupling strength in twisted bilayer graphene could be further modified by changing twist angle, which makes twisted bilayer graphene more interesting 2DES for studying various correlation-driven QH states.

## Methods

### Device fabrication

The top h-BN was exfoliated onto double-layer spin coated polymer stack, consisting of 1μm polypropylene carbonate (PPC) and poly(4-styrenesulfonic acid) layer on top of a bare Si substrate. As dry transfer method^13^, we transferred h-BN/PPC film to PDMS/Glass stamps. Using micromanipulator, we picked up the top and bottom graphene. Finally, the stack was released to h-BN on top of the SiO_2_/Si substrate. In order to make Hall bar geometry, the h-BN and graphene were etched by CF_4_ and O_2_ plasma with PMMA etching mask. Then, electrode was patterned by e-beam lithography. For decreasing contact resistivity and getting fresh edge, we re-treated CF_4_ and O_2_ plasma just before metal evaporations. Gold electrodes (70 nm) were evaporated with Cr adhesion layer (10 nm). The base pressure in e-beam chamber was 8 × 10^−8^ mbar.

### Transport measurements

Transport measurements have been performed with standard lock-in technique (excitation frequency *f* = 17.777 Hz and current *I* = 100 nA) using a physical property measurement system (Quantum Design, PPMS) and an superconducting magnet at the National High Magnetic Field Laboratory, USA.

### Raman spectroscopy measurements

Raman spectra were measured by a Nd:YAG laser operating at a wavelength of 532 nm and laser power of 0.1 mW (XperRam200/Nanobase and Alpha 300 R/WITEC) at room temperature (*T*~293 K) under ambient conditions.

## Additional Information

**How to cite this article**: Kim, Y. *et al*. Broken-Symmetry Quantum Hall States in Twisted Bilayer Graphene. *Sci. Rep.* **6**, 38068; doi: 10.1038/srep38068 (2016).

**Publisher's note:** Springer Nature remains neutral with regard to jurisdictional claims in published maps and institutional affiliations.

## Supplementary Material

Supplementary Information
