# Supplementary material for: Broken-Symmetry Quantum Hall States in Twisted Bilayer Graphene
Source: Sci Rep. 2016 Dec 1;6:38068. doi: 10.1038/srep38068 (PMC5131475; doi:10.1038/srep38068)
Supplement: Supplementary Information [file srep38068-s1.pdf]

Supplementary Information

Broken-Symmetry Quantum Hall States in Twisted Bilayer Graphene

Youngwook Kim[1†], Jaesung Park[2], Intek Song[3,4], Jong Mok Ok[1], Younjung Jo[5], K. Watanabe[6], T. Taniquchi[6], Hee Cheul Choi[3,4], Dong Su Lee[7], Suyong Jung[2], and Jun Sung Kim[1*]

[1]Department of Physics, Pohang University of Science and Technology, Pohang 37673, Korea

[2]Korea Research Institute of Standards and Science, Daejeon 305-340, Korea

[3]Center for Artificial Low Dimensional Electronic System, Institute for Basic Science (IBS), Pohang 37673, Korea

[4]Department of Chemistry, Pohang University of Science and Technology, Pohang 37673, Korea

[5]Department of Physics, Kyungpook National University, Daegu 702-701, Korea

[6]National Institute for Materials Science, 1-1 Namiki, Tsukuba 305-0044, Japan

[7]Applied Quantum Composites Research Center, KIST Jeonbuk Institute of Advanced Composite Materials, Jeonbuk 55324, Korea

*e-mail: js.kim@postech.ac.kr

[†]Present address: Max-Planck-Institut für Festköperforschung, 70569, Stuttgart, Germany.

S1. Raman Spectroscopy of twisted bilayer graphene

In order to characterize the twist angle of our device, we used Raman spectroscopy, which has been widely employed to determine the twist angle (θ) in twisted bilayer graphene. As reported in the previous Raman studies on twisted bilayer graphene,[39-41] the characteristic features of G and 2D modes in the Raman spectra show strong dependence on the twist angle. Figure 1(a) shows the Raman spectra taken at the monolayer and the twisted bilayer regions in the device 1 (D1). Similar results were also obtained in the device 2 (D2). The corresponding optical image, before electrode patterning, is shown in Fig. 1(i). We found that the G peak is comparable in intensity with the 2D peak in twisted bilayer, in strong contrast to the monolayer case. Also the 2D peak is blue shifted by ~ 9 cm^{-1} compared to that from single-layer graphene.

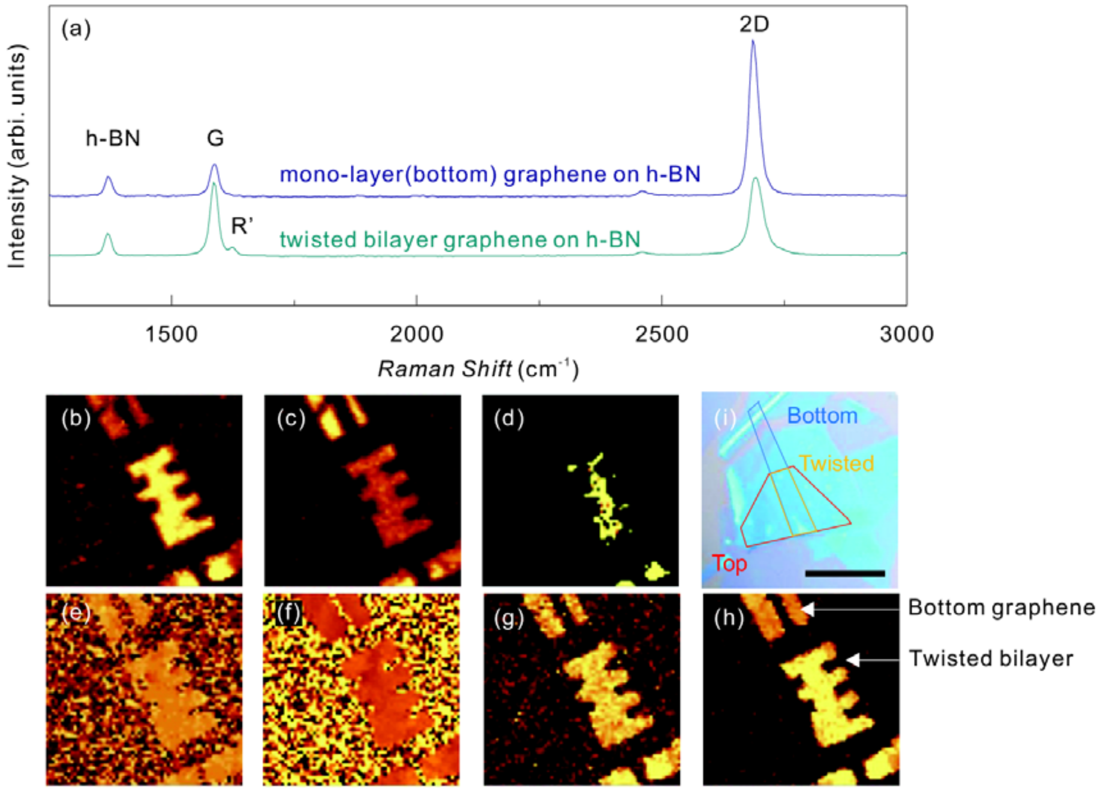

Figure S1. (a) Raman spectra of twisted bilayer and monolayer graphene in the device 1 (D1). Raman mapping of (b) G peak intensity, (c) 2D peak intensity, (d) R' peak intensity, (e) G peak position, (f) 2D peak position, (g) G peak FWHM and (h) 2D peak FWHM. (i) Optical microscope image of the sample with 10 μm scale bar.

Additional Raman peak next to the G mode is observed in the twisted bilayer graphene, which is known as the *R*' mode at 1618 cm^{-1}, one of the characteristic Raman mode observed only in twisted bilayer graphene.[41] Strong enhancement of the R' mode taken at different excitation laser of 633 nm is consistent with previous report[41] (Fig. 2(f)).

In Figs. S2(a)-(d), we plot the four characteristic features in G and 2D modes, *i.e.*, the intensities of 2D and G modes, the relative shift of 2D Raman mode with respect to that in monolayer, and the full-width-at-half-maximum (FWHM) of 2D peak, taken at our devices D1 and D2 together with previous results on samples with various twist angles.[39,40] From comparison, we found that the twist angle is estimated to be ~ 5° for the D1 and ~ 3° for the D2 . This angle is further confirmed by the observed R' mode. The R' mode is known to be sensitive

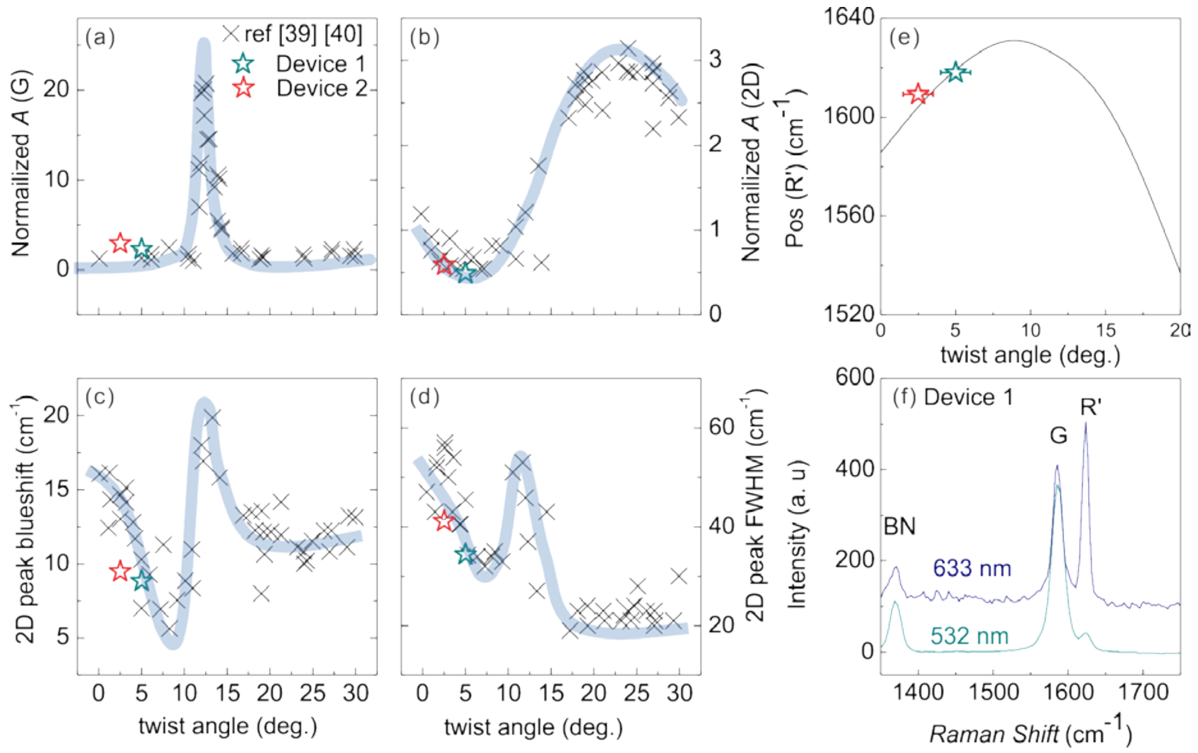

Figure S2. Normalized intensities of (a) G and (b) 2D peaks, (c) relative shift of the 2D peak with respect to the monolayer case, and (d) FWHM of the 2D peak in the twisted bilayer graphene. The data taken from our samples are compared with those in Refs. 39 and 40. (e) The position of the R' Raman mode as a function of twist angle. The black solid line is theoretical data (Ref. 41) and the dot is experimental data. (f) Raman spectra near the R' peak at different excitation energy for the device 1.

to moiré potential and thus can be used to extract the twist angle in twisted bilayer graphene. The peak position of the R’ mode in our sample is consistent with the twist angle of either ~5°(3°) or ~ 15° (18°) for the D1 (D2) when compared with theoretical calculations[41] (Fig. S2(e)) . The latter however cannot explain the other features of G and 2D modes shown in Figs. S2(a)-(d). Based on these results, we can conclude that our twisted bilayer graphene has its twist angle of ~ 5° for the D1 and ~ 3° for the D2. Furthermore, all mapping images shown in Figs. S1(b)-(h) indicate that our twisted bilayer is homogeneous.

For a twisted bilayer graphene with a twist angle 5°(3°) for the D1 (D2), the corresponding moiré period is ~ 3.6 (4.0) mm, and Fermi velocity is expected to be reduced by 15% (25%) with respect to that of monolayer graphene. The van Hove singularity point is expected to be located at ~ 600 (300) meV above the Dirac point. This is far beyond the accessible chemical potential by the back-gate field across a 290 nm-thick dielectric layer. Thus, in our experiments, the twist bilayer graphene is in the large angle regime.

S2. Screening and charge redistribution in twisted bilayer graphene

Due to the low density of the states of graphene, external electric field from back-gate voltage (V_g) penetrates the lower layer graphene of twisted bilayer graphene, leading to charge redistribution between the layers. The total induced charge carrier density, *i.e.* the summation of the charge densities of upper and lower layers is given by

$$eV_g = e^2(n_L + n_U)/C_{dielectric} + \mu(n_L) \text{ ...(a)}$$

, where $C_{dielectric}$ is the capacitance of the insulating layer under twisted bilayer graphene, and $\mu(n_L)$ is the chemical potential of the lower layer. The capacitance $C_{dielectric}$ is expressed by $C_{dielectric} = \varepsilon_{dielectric}\varepsilon_0/d$, where d is the layer separation between the back-gate to graphene. Since

the dielectric constant of *h*-BN is similar to SiO_2 ($\varepsilon_{dielectric}$ ~3.9), we used d ~ 290 nm, considering both 270-nm-thick SiO_2 and 20-nm-thick h-BN. The upper layer density is defined by $n_U = \varepsilon_{GG} \cdot F_L / e$, where $F_L = V_L / d_{GG}$ is electric field between the layers, ε_{GG} is dielectric constant in twisted bilayer graphene, and d_{GG} ~ 0.4 nm is layer separation of twisted bilayer graphene. Then the chemical potentials with respect to the charge neutral point of upper and lower graphene layers, $\mu(n_U)$ and $\mu(n_L)$ are described by

$$eV_{res} = \mu(n_L) - [\mu(n_U) + e^2 n_U / C_{GG}] \text{ ...(b)}$$

, where eV_{res} is extrinsic electric field by residual charges and C_{GG} is capacitance between twisted bilayer. Using the equations above, we can calculate the n_U and n_L with a variation of V_g as shown in Fig. 2(d) of the main text. Here, we ignore the chemical potential term of the lower layer, $\mu(n_L)$, in the equation (a). In principle, both the charging energy term, $e^2(n_L + n_U)/C_{dielectric}$, and the chemical potential term, $\mu(n_L)$, depend on the gate voltage V_g. However in our devices with the charge neutral point at $V_g \approx 0$ V, as shown in Fig. 1 of the main text, the gate-field induced change is dominated by the charging energy term $e^2(n_L +$

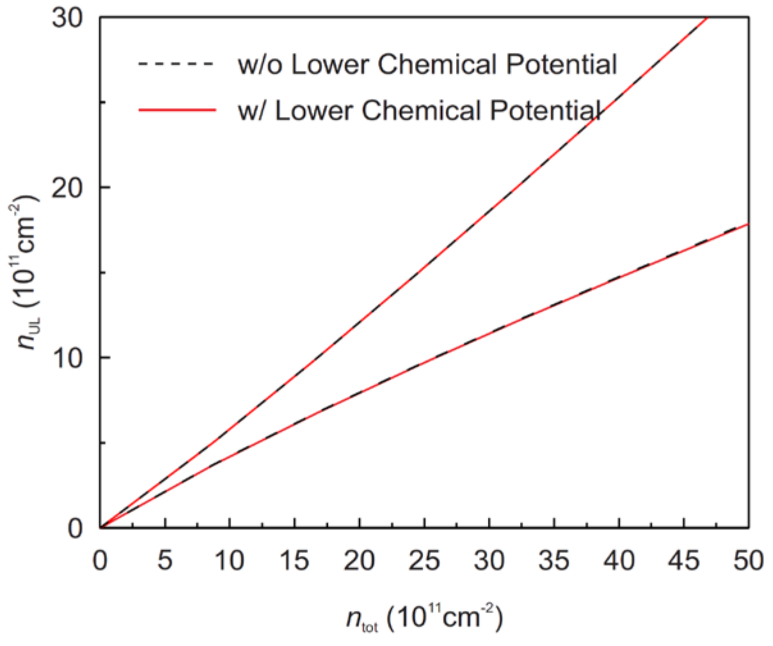

Figure S3. The calculated upper and lower layer carrier density, n_U and n_L, as function of total carrier density with (red) and without (black dot) taking the lower layer chemical potential term in the equation (a) (see the text).

$n_U)/C_{dielectric}$, rather than the chemical potential term $\mu(n_L)$. In the whole range of V_g of our experiments, $\mu(n_L)$ varies by only 1% of the change in the charging energy. For example, with n_L ~ 10^{12}/cm^2, one can obtain $\mu(n_L)$ ~ 100 meV, whereas the charging energy $e^2(n_L + n_U)/C_{dielectric}$ ~ 10 eV. Therefore, with or without taking $\mu(n_L)$ into account, the calculated carrier densities of the upper and lower layers, n_U and n_L, show almost identical V_g dependences as shown in Fig. S3. Thus, we neglect $\mu(n_L)$ term in Eq. (a) for calculations. Note that this approach, neglecting $\mu(n_L)$ in Eq. (a), has been successfully employed in, *e.g.*, Ref. 32 of the main text.

S3. Charge filling to upper and lower layer in presence of magnetic field

Under magnetic fields, we first construct the Lorentzian-shape density of states (DOS) for LLs with a peak broadening of 0.01 meV at $E_N = \mathrm{sgn}(N)v_F(2e\hbar B|N|)^{1/2}$. Then using equations (a) and (b) above, V_g dependences of n_U and n_L are calculated at a given magnetic field. We used the interlayer dielectric constant $\varepsilon_{GG} = 2.45\varepsilon_0$ (ε_0 is the permittivity of vacuum) and the Fermi velocity of $v_F = 0.85 \times 10^6$ m/s (D1) and $v_F = 0.75 \times 10^6$ m/s (D2) that are estimated from Raman spectroscopy shown in Fig. S2. In order to reproduce the observed electron-hole asymmetry, we introduced V_{res} = 7 mV (4 mV) for D1 (D2), corresponding to the residual charges with a density of 2.0×10^{11} cm^{-2} (1.0×10^{11} cm^{-2}) as obtained from Fig. 1(a) in the main text.

The calculated carrier density for each layer is presented in the left upper panel of Fig. S4 as a function of total filling factor at B = 10 T. Here we only consider the normal integer QH states without lifting spin and valley degeneracy. The schematic illustrations from **a** to **j** represent charge filling of the Landau level (LL) in the upper and the lower layers at each point marked in the left upper panel of Fig. S4. Due to the screening of the gate field by the lower layer, filling rates between the upper and the lower layers are expected to be different. This is simply described in schematic illustration by larger distance between the LLs in the upper layer

than in the lower layer.

At the configuration **a**, the LL of the upper layer with the Landau index N_U = -1 is already filled, while the gate-induced charges enter into the LL of the lower layer (N_L = -1). After the N_L = -1 level is filled, both layers become incompressible in the configuration **b** (ν_{tot} = -4). With further increasing V_g, the charging energy blocks the gate-induced electrons entering into the N_U = 0 level, and instead the N_L = 0 level starts to be filled up in **c**. In this case, before the N_L = 0 state is completely filled, the N_U = 0 level starts to be filled up in **d**. Then both N_L = 0 and N_U = 0 LLs are simultaneously filled until both layers become incompressible in **e** (ν_{tot} = +4). Similarly one can follow the sequences of the filling of each layer from **f** to **j** between the

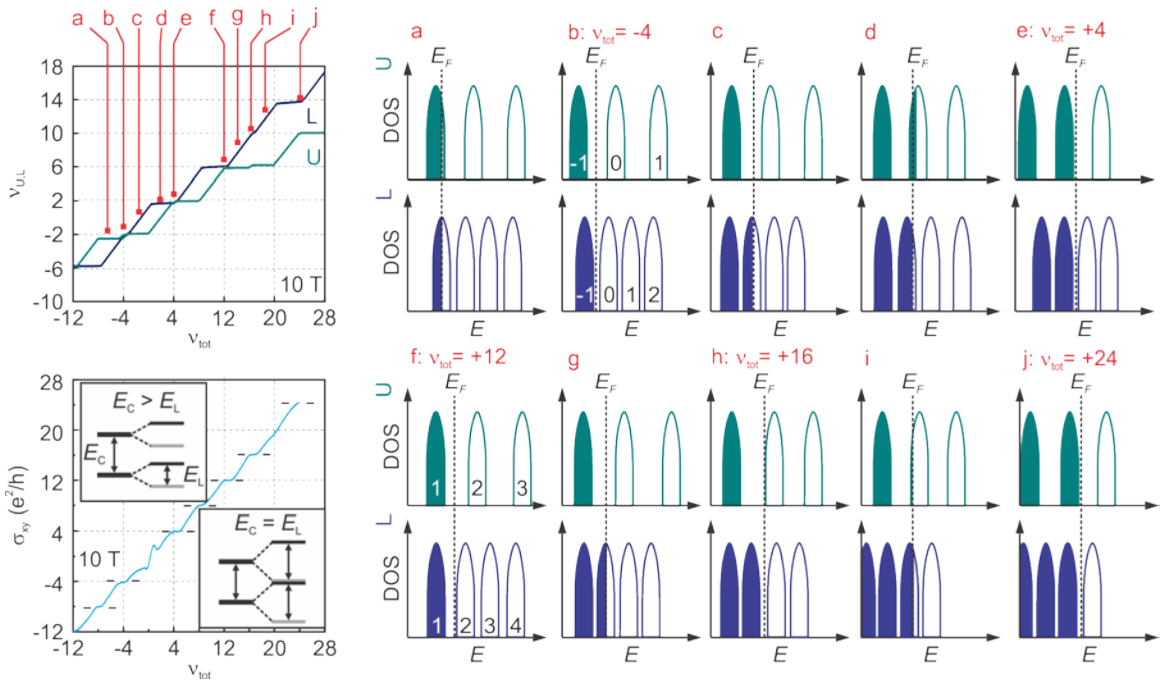

Figure S4. (Left upper) individual upper and lower layer charge carrier density obtained through model calculation at the 10 T. Navy (dark cyan) represent lower(upper) layer carrier density. (Left lower) Experimentally observed quantum Hall curve at 10 T in D1. Inset display parent quantum Hall gap is changed by increasing layer polarized energy gap, even itself is closed when the layer polarized energy gap is equal to cyclotron gap. (Right) Schematic illustrations for charge filling of upper and lower Landau level. The letters correspond to the data marked in Fig.S4 left upper panel.

QH states of ν_{tot} = +12 and ν_{tot} =+24. Here, after filling of the N_U = 1 level in **f**, two LLs with N_L = 2 and 3 in the lower layer are successively filled in **g** and **h**. In this case, the LLs of N_L = 3 and N_U = 2 are filled together in **i** until the ν_{tot} =+24 state in **j**. Note that in some cases (**d** and **i**), two LLs in both layers are filled together. This occurs when the cyclotron energy becomes comparable with the layer polarization gap induced by the displacement field as shown in the lower left panel of Fig. S4. As a result, one of the layers remains compressible across the ν_{tot} = 0 (**d**) and ν_{tot} = 20 (**j**) states and the corresponding QH plateau is expected to be missing. This is indeed what experimentally observed as shown in the lower left panel of Fig. S4.

S4. Shubnikov-de Haas oscillations in the high V_g region

Figure S5 shows Shubnikov-de Haas (SdH) oscillations as a function of the inverse magnetic fields, taken from D1 at three representative total carrier densities. The observed SdH oscillations contain multiple frequencies, reflecting two different Fermi surfaces in size from the lower and the upper layers. In the high V_g region (high total carrier density), the displacement field introduces charge imbalance, and two different SdH frequencies are

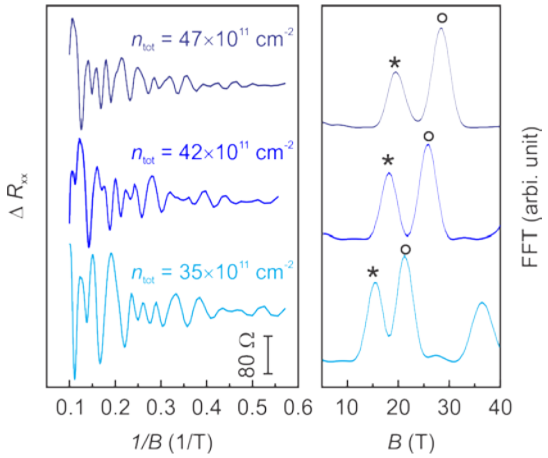

Figure S5. (Left) Shubnikov-de Hass oscillation with different total carrier density at the 1.8 K (Right) Fourier transformation results from left panel. Three different color curves indicate different total charge density. Each symbol (star and circle) represent upper and lower layer carrier density.

expected in the fast Fourier transformed spectra. This is indeed the case in experiments as shown in Fig. S5. From the observed SdH frequency (F_{SdH}), we extracted the carrier densities of the upper (n_{U}) and the lower (n_{L}) layers using the relations; $F_{\mathrm{SdH}} = ge/nh$, where the degeneracy factor g = 4 (2-spin and 2-valley), e is elementary charge, and h is plank constant. The extracted carrier densities are plotted in Fig. 2(d) in the main text, together with the calculated density curves as discussed in the section S2. Excellent agreement between experiments and calculations demonstrate that the filling of LLs in twisted bilayer graphene is well captured by the calculations taking the gate-field induced charge imbalance into account.

S5. Weak $\nu = 0$ ($\sigma_{xy}= 0$) state twisted bilayer graphene

In monolayer graphene, diverging R_{xx} (>100 kΩ) is observed for the $\nu = 0$ ($\sigma_{xy}= 0$) state due to the absence of the edge channels.[24,37] This is a consequence of the characteristic hierarchy of symmetry breaking; valley degeneracy is first lifted, and then spin degeneracy is lifted. In twisted bilayer graphene, if considered as two monolayer graphene, one can expect the similar diverging R_{xx} due to the absence of edge channels. However, as discussed in the main text, the layer hybridization opens a gap Δ_{SAS} in the $\nu_{\mathrm{tot}} = 0$ state and the crossover between the broken-symmetry states with different configurations leads to a resistivity deep at B ~ 10 T as shown in Fig. 3(f) in the main text. This may explain why the $\nu_{\mathrm{tot}} = 0$ state shows relatively low resistivity (<10 kΩ) and weak signature of the σ_{xy} plateau, on contrary to the case of monolayer graphene. Above the transition field at B ~ 10 T, R_{xx} rapidly diverges with increasing magnetic field and reaches up to ~ 40 kΩ at B = 14 T. Also a hint of the σ_{xy} plateau is observed as shown in Fig. 3d of the main text. Therefore, in twisted bilayer graphene, one can expect that the σ_{xy} =0 plateau becomes visible at relatively higher magnetic fields than in

monolayer graphene, if assumed the same device quality. As found in Ref. 35 of the main text, the clear σ_{xy} = 0 plateau appears at B = 35 T in monolayer graphene when R_{xx} reaches ~ 150 kΩ, which is 4 times larger than observed in our devices. Therefore, we expect that the σ_{xy} = 0 would appear at higher magnetic fields, far above the transition field ~ 10 T.

S6. Gap energies of the broken-symmetry quantum Hall states

We estimated the size of energy gaps for each broken-symmetry quantum Hall states from the temperature dependence of R_{xx}. For D1, the minima of R_{xx} for ν_{tot} = ±1, ±2, and ±3 are taken as a function of temperature at different magnetic fields from 11 to 14 T, which is fitted to the Arrhenius formula as shown in Fig. S3 (a)-(f). Similarly we also estimated the transport gap

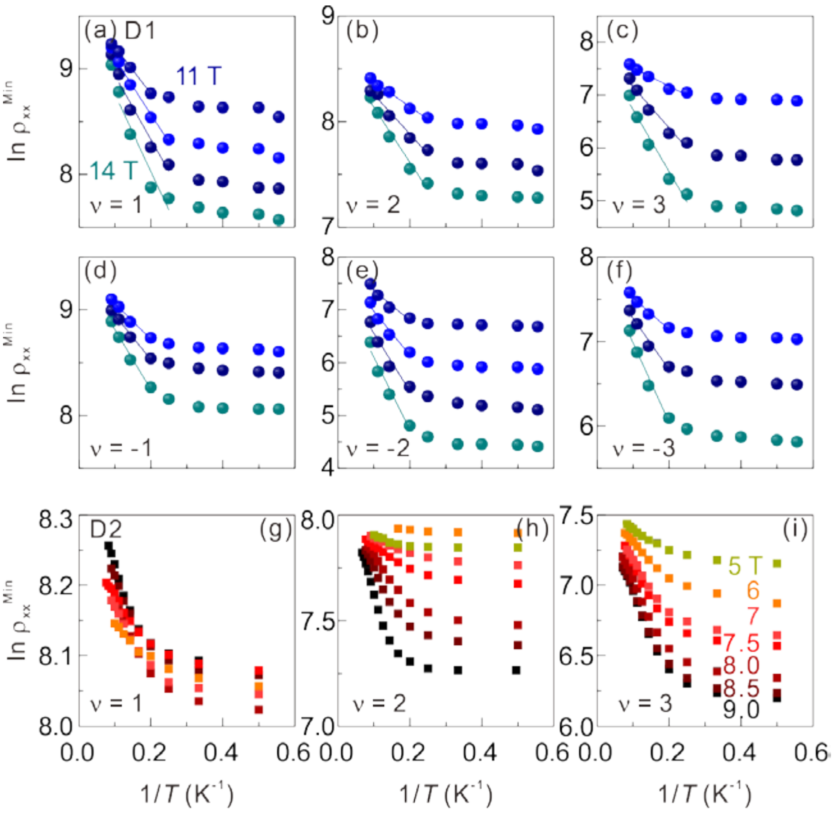

Figure S6. Arrhenius plot for the QH states with (a) ν_{tot} = 1, (b) 2, (c) 3, (d) –1 (e) –2 and (f) –3, taken from D1 at different magnetic fields from 11 to 14 T in 1 T steps. Arrhenius plot for the QH states with (g) ν_{tot} = 1, (h) ν_{tot} = 2, and (i) ν_{tot} = 3, taken from D2.

for the QH states with ν_{tot} = 1, 2, and 3 as shown in Fig. S3 (g)-(i). Note that in D2 the temperature dependence of R_{xx} at B = 6 T is distinct from those taken at different magnetic fields.

References

39. Kim, K. *et al.* Raman Spectroscopy Study of Rotated Double-Layer Graphene: Misorientation-Angle Dependence of Electronic Structure. *Phys. Rev. Lett.* **108**, 246103 (2012).

40. Havener, R. W., Zhuang, H., Brown, L., Hennig, R. G. & Park, J. Angle-resolved Raman imaging of interlayer rotations and interactions in twisted bilayer graphene. *Nano Lett.* **12**, 3162–3167 (2012).

41. Carozo, V. *et al*. Raman signature of graphene superlattices. *Nano Lett.* **11**, 4527–4534 (2011).
